# Optimization of Extraction of Phlorotannins from the Arctic *Fucus vesiculosus* Using Natural Deep Eutectic Solvents and Their HPLC Profiling with Tandem High-Resolution Mass Spectrometry

**DOI:** 10.3390/md21050263

**Published:** 2023-04-23

**Authors:** Ekaterina D. Obluchinskaya, Olga N. Pozharitskaya, Vadim A. Shevyrin, Elena G. Kovaleva, Elena V. Flisyuk, Alexander N. Shikov

**Affiliations:** 1 Research Group of Biochemistry and Technology of Hydrobionts of Algae and Invertebrates, Murmansk Marine Biological Institute of the Russian Academy of Sciences (MMBI RAS), 17 Vladimirskaya Str., 183010 Murmansk, Russia; obluchinskaya@gmail.com (E.D.O.); olgapozhar@mail.ru (O.N.P.); 2 Scientific, Educational and Innovation Center of Chemical and Pharmaceutical Technologies, Ural Federal University Named after the First President of Russia B. N. Yeltsin (UrFU), 19 Mira Str., 620002 Ekaterinburg, Russia; v.a.shevyrin@urfu.ru (V.A.S.); e.g.kovaleva@urfu.ru (E.G.K.); 3 Department of Technology of Pharmaceutical Formulations, St. Petersburg State Chemical Pharmaceutical University, 14a Prof. Popov Str., 197376 Saint Petersburg, Russia; elena.flisyuk@pharminnotech.com

**Keywords:** Arctic, alga, seaweed, *Fucus vesiculosus*, extraction, natural deep eutectic solvent, NADES, phlorotannins, response surface methodology (RSM)

## Abstract

Phlorotannins are secondary metabolites produced mainly by brown seaweeds (Phaeophyceae) and belong to the class of polyphenolic compounds with diverse bioactivities. The key factors in the extraction of polyphenols are the selection of a suitable solvent, method of extraction and selection of optimal conditions. Ultrasonic-assisted extraction (UAE) is one of the advanced energy-saving methods suitable for the extraction of labile compounds. Methanol, acetone, ethanol and ethyl acetate are the most commonly used solvents for polyphenol extraction. As alternatives to toxic organic solvents, a new class of green solvents, natural deep eutectic solvents (NADES), has been proposed for the efficient extraction of a wide range of natural compounds including polyphenols. Several NADES were screened previously for the extraction of phlorotannins; however, the extraction conditions were not optimized and chemical profiling of NADES extract was not performed. The purpose of this work was to study the effect of selected extraction parameters on the phlorotannin content in NADES extract from *Fucus vesiculosus,* optimization of extraction conditions and chemical profiling of phlorotannins in the NADES extract. A fast and green NADES-UAE procedure was developed for the extraction of phlorotannins. Optimization was performed through an experimental design and showed that NADES (lactic acid:choline chloride; 3:1) provides a high yield (137.3 mg phloroglucinol equivalents per g dry weight of algae) of phlorotannins under the following extraction conditions: extraction time 23 min, 30.0% water concentration and 1:12 sample to solvent ratio. The antioxidant activity of the optimized NADES extract was equal to that of EtOH extract. In total, 32 phlorotannins have been identified (one trimer, two tetramers, six pentamers, four hexamers, six heptamers, six octamers and seven nonamers) in NADES extracts from arctic *F. vesiculosus* using the HPLC-HRMS and MS/MS technique. It was noted that all the above-mentioned phlorotannins were identified in both EtOH and NADES extracts. Our results suggest that NADES could be considered as an alternative to the conventional techniques for the effective extraction of phlorotannins from *F. vesiculosus* with high antioxidant potential.

## 1. Introduction

*Fucus vesiculosus* L. (Phaeophyceae) is a brown seaweed widely distributed in the Arctic region and considered to be a good source of fucoidan, alginates, laminarans and polyphenols [1,2]. Polyphenols refer to chemical compounds (monomeric, oligomeric or polymeric) formed by an aromatic ring with one or more hydroxyl substituents [3].

Phlorotannins are secondary metabolites produced mainly by brown seaweeds (Phaeophyceae), and belong to the class of polyphenolic compounds [4,5]. The biological activities of phlorotannins from marine brown seaweeds have been explored extensively [6]. Antiviral [7], anti-bacterial [8], antioxidant [9], anticancer [10], anti-inflammatory [11], neuroprotective [12], UV-protective [13], etc. activities have been reported for seaweed phlorotannins. Phlorotannins have a wide range of applications in several industries. They are constituents of sunscreens as anti-aging and UV-protective agents [14,15]. Due to their antimicrobial and antioxidant properties, phlorotannins are used in food packaging films as preservatives [16]. Often, phlorotannins are co-extracted with fucoidan, and provide advantageous cosmeceutical effects for fucoidan crude extracts [17]. Phlorotannins are approved in the European Union as new food products and are allowed as ingredients in dietary supplements [18].

**Figure 1:**
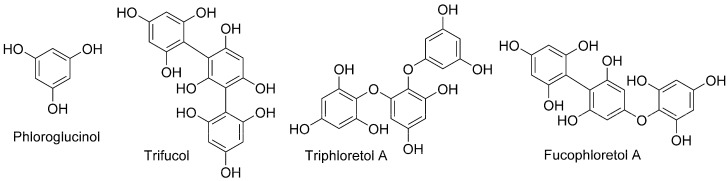
The main phlorotannins according to the structural units.

The pharmacological activity of phlorotannins depends on their structure and the degree of polymerization of the structural units (1,3,5-trihydroxybenzene) of phloroglucinol. In general, phlorotannins have been classified into fucols (with only phenyl linkages), phlorethols (phlorotannins with only aryl ether bonds) and fucophlorethols (with aryl ether and phenyl linkages) (Figure 1). The structural diversity rises with increasing numbers of phloroglucinol units, and they can be grouped into linear phlorotannins, two inter-phloroglucinol connections or branched compounds [19].

The key factors for the extraction of polyphenols are the selection of a suitable solvent, method of extraction [20], and the fixing of optimal conditions (temperature, process time, extraction modulus, etc.), which directly affect the efficiency of the process and the yield of active substances [18]. Different methods including maceration [21], microwave-assisted extraction [22], pressurized liquid extraction [23], supercritical fluid extraction [24], ultrasonic-assisted extraction (UAE) [25], etc. have been employed for phlorotannin separation. UAE is one of the advanced energy-saving methods suitable for the extraction of labile compounds [26]. In this process, ultrasonic waves create cavitation bubbles, which, when colliding, provide a hot-spot/microjet of higher temperature and pressure. This results in faster and more efficient extraction of active compounds from a plant matrix [27,28]. The yield and quality of the extract significantly depend on a number of UAE factors [29], such as the power/intensity of ultrasound, the form of the extraction vessel, the temperature and time of extraction and the solvent, its concentration and volume [27,30,31,32]. UAE has been widely implemented and optimized for the extraction of polyphenols from seaweed [25,32,33,34].

Methanol, acetone (70%), ethanol and ethyl acetate are the most commonly used solvents for polyphenols [4,18]. As an alternative to toxic organic solvents, a new class of green solvents, natural deep eutectic solvents (NADES), have been applied in sample preparation and analytical techniques [35] for the efficient extraction and separation of a wide range of natural compounds [36] including polyphenols [37,38,39]. UAE was used to increase the efficiency of NADES in the extraction of phenolic compounds [40,41]. UAE with NADES is considered a green technology due to its advantages of high extraction efficiency, low solvent, energy and effective time consumption [40,42].

The optimization of parameters affecting the efficiency of UAE is important to obtain the maximum yield of the target compounds. Response surface methodology (RSM) is an efficient optimization technique that requires reasonable experimental trials to evaluate multiple parameters and their interactions [43]. RSM is used to design improved and optimized processes where the response of interest (e.g., content of active compounds) is influenced by multiple variables, and the goal is to optimize the response. Thus, experimental design approaches including Box–Behnken design (BBD), RSM and the steepest ascent/descent method are used to obtain more information from a limited number of experiments.

Recently, phlorotannins from *F. vesiculosus* were extracted with NADES [44]. However, the individual phlorotannins were not identified.

In this context, we aimed to study the effect of selected extraction parameters on the phlorotannin content of NADES extract from *F. vesiculosus* collected in the Arctic, and to optimize this process using BBD and RSM, which use quantitative data from the corresponding experiment design to determine or simultaneously solve a multivariate equation. The antioxidant activity of the optimized NADES extract will be compared with that of the EtOH extract. Finally, the chemical profiling of phlorotannins of the NADES extract from *F. vesiculosus* will be performed using high-performance liquid chromatography–high-resolution mass spectrometry (HPLC-HRMS) with tandem (MS/MS) experiments.

## 2. Results and Discussion

Recently, much attention has been paid to the selection of evaluation criteria for extraction greenness of the proposed method based on 10 impact categories including the choice and use of solvents, materials and reagents, waste generation, energy consumption, sample size and throughput, among others [45]. The determination of significant technological factors and the optimization of the extraction process are crucial for obtaining a high-quality extract. UAE was applied for the extraction of phlorotannins from *F. vesiculosus.*

### 2.1. The Ultrasound-Assisted Extraction of Phlorotannins from F. vesiculosus with NADES

Based on the literature data [25,29] and the results of our preliminary studies [44], the extraction time, the concentration of water in a solvent and the solvent–solid ratio were selected as factors affecting the yield of total phlorotannins (TPhC) in the NADES extract (Table 1). NADES lactic acid:choline chloride (LA:ChCl) 3:1 was chosen based on the criteria for extraction greenness as safe [46] and synergistic [47] to optimize the yield of phlorotannins from brown algae.

**Figure 2:**
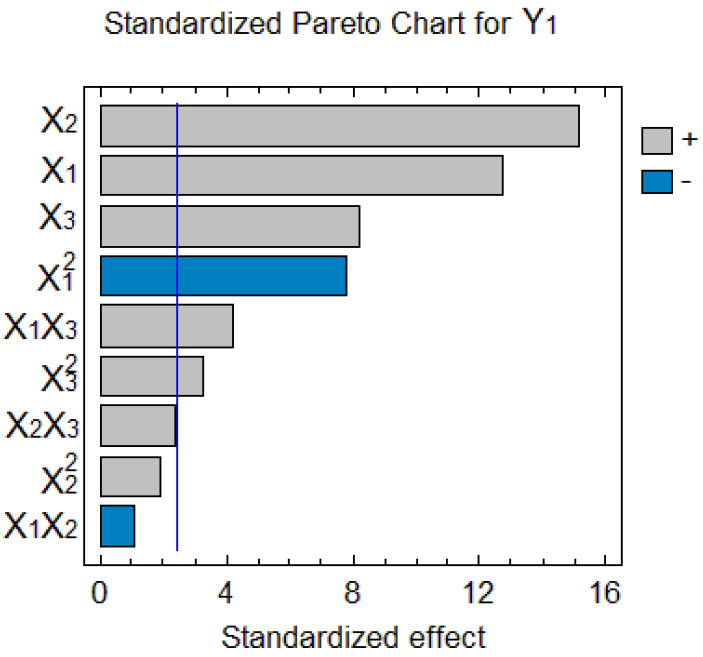
Pareto chart of the variables’ effects on the TPhC.

Table 2 shows the experimental conditions for the BBD with 15 runs, as well as TPhC results under the conditions evaluated. The total phlorotannin content in the NADES extracts of *F. vesiculosus* varied from 22.0 to 98.2 mg TPhC/g DW. The results were statistically analyzed, and Figure 2 presents the Pareto chart with the independently studied factors in terms of TPhC. It is clear from this figure that under the evaluated experimental conditions, all three factors showed a significant effect (*p* < 0.05) on TPhC.

The water concentration in NADES showed the most significant effect on the yield of TPhC from *F. vesiculosus*. The effect was clearly observed when comparing the results of experiments 4 and 6, as well as the yield reported in experiments 15 and 14. The TPhC rose with increasing water concentration in NADES and solid to solvent ratio due to a higher driving force in the more diluted solution [48]. Diffusion is favored by an increased amount of the solvent in contact with a sample and by a decrease in viscosity [49], which results in the increased concentration gradient within the solid matrix and more efficient extraction.

### 2.2. Model Adjustment and Analysis of Variance

The experimental data presented in Table 2 were statistically assessed, and the coded model coefficients and *p* values for TPhC are presented in Table 3. From the data regression analysis, it was found that the TPhC and the experimental variables are correlated according to Equation (1) (*p* = 0.007 < 0.0):

TPhC = 60.3 + 13.3X_1_ + 15.8X_2_ + 8.5X_3_ − 11.5X_1_^2^ + 2.9X_2_^2^ + 4.8X_3_^2^ − 1.6X_1_X_2_ + 6.2X_1_X_3_ + 3.5X_2_X_3_ (1)

The validity of the predictive equation was verified using an *F*-test and analysis of variance (ANOVA). The model is validated, or has a satisfactory fit to the experimental data, when *F_CALC_* > *F_TAB_* (calculated from the ANOVA table and tabulated, respectively). The correlation coefficient value (0.989) and the *F*-test for regression showed that the model was capable of representing the experimental data in the range of factors investigated. The statistical analysis presented values of 62.23 and 4.10 for *F_CAL_* and *F_TAB_*, respectively. The results of the ANOVA analysis of each response are presented in Table 3. The model developed for TPhC was significant at *p* < 0.0002 and had a satisfactory *R*^2^ and adjusted *R*^2^ (0.989 and 0.970, respectively). These parameters of the model developed were very close. Thus, there was a good conformity between the experimental and predicted values, proving that they can be used for the prediction and optimization stages [50].

The model (Equation (1)) shows that all three variables selected as independent factors affecting TPhC extraction from *F. vesiculosus* were significantly linear, influencing TPhC with *F* values in the range of 191.84–56.20 mg PhE/g DW (*p* < 0.0007), which means they are required for a complete understanding of the behavior of this response. The linear values of extraction time and solvent ratio also had significant effects on TPhC. The interaction of the linear units of extraction time and molar ratio had significant impacts on TPhC with an F value of 14.65 (*p* < 0.0123). Likewise, the quadratic terms of the extraction time and molar ratio had the most significant impact on TPhC, with *F* values of 46.64 and 8.22 (*p* < 0.0010 and *p* < 0.0351, respectively) (Table 3).

### 2.3. Response Surface Methodology and Optimization

**Figure 3:**
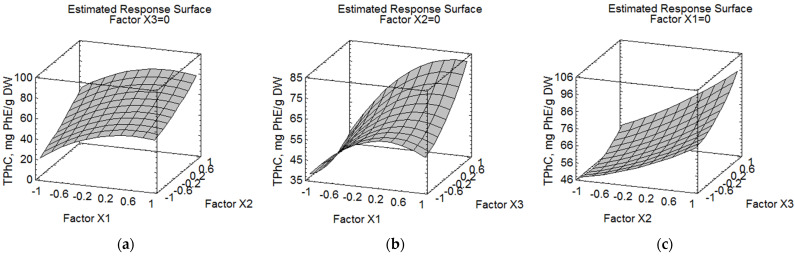
Response surface and contour plots for the total phlorotannin content (TPhC, expressed as mg of phloroglucinol equivalents/g of dried algae, mg PhE/g DW) from *F. vesiculosus* extracts with respect to (**a**) water concentration (%, X_2_) and extraction time (min, X_1_), (**b**) extraction time (min, X_1_) and sample to solvent ratio (m/m, X_3_) and (**c**) water concentration (%, X_1_) and sample to solvent ratio (m/m), X_3_). The third variable of each graph was kept at its zero level.

The 3D response surface and contour plots (Figure 3a–c) illustrate the relationship between total phlorotannin content in the *F. vesiculosus* extract and experimental variables of extraction time, water concentration and sample to solvent ratio. The steepest ascent/descent method was applied for the calculation of optimal extraction conditions [43]. The ideal circumstances that were chosen for the optimized parameters using the created models above are presented in Table 4.

We performed verification experiments under the selected conditions with two repetitions to confirm the fitness of the predicted response values. A slight difference was observed between the predicted TPhC content of both responses and the experimental results (Table 4), which proved the fitness of the design used for determining the optimized processing conditions with the desired results.

Recently, G.A. Sumampouw et al. (2021) reported that a maximum TPhC of 36.9 PhE/g DW was found in the EtOH extract (58.65% *v*/*v*) of *F. vesiculosus* obtained through pressurized liquid extraction at a temperature of 137 °C [51]. The maximal TPhC in the optimized acetone 67% (*v*/*v*) extract of *F. vesiculosus* was 36.9 mg gallic acid equivalent/g DW, achieved at a solvent to solid ratio of 70 mL/g and temperature of 25 °C [52]. In another experiment, the optimized conditions for the extraction of phlorotannins from brown seaweed *Sargassum swartzii* were EtOH (52% *v*/*v*) and a solvent/seaweed ratio of 33:1 (*v*/*w*). The TPhC content reached 55.9 mg PhE/g DW [53]. In our previous study, we applied 10 different NADES for the extraction of phlorotannins from *F. vesiculosus* and *Ascophyllum nodosum*. The TPhC was varied from 8.7 to 61.2 mg PhE/g DW and from 7.8 to 50.3 mg PhE/g DW for *F. vesiculosus* and *A. nodosum*, respectively, after maceration with stirring for 2 h at 50 °C [45]. In the current study, the optimization of the UAE-assisted extraction of phlorotannins from *F. vesiculosus* allowed a significant increase in the yield of phlorotannins in the NADES extract (Table 4). Our results suggest that NADES can effectively replace organic solvents for the extraction of phlorotannins from seaweed.

### 2.4. Antioxidant Activity of Optimized Extract

**Figure 4:**
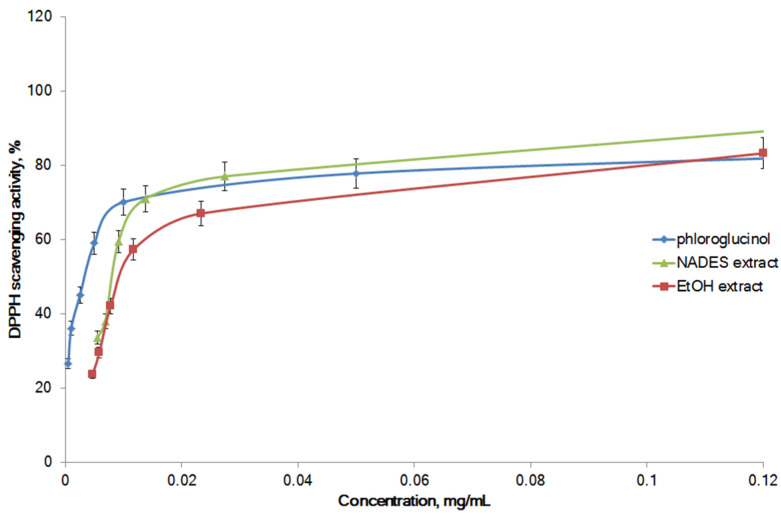
DPPH radical scavenging activity for *F. vesiculosus* extracts. Each value represents the mean ± SD of three trials.

Commonly, the antioxidant potential of seaweed extracts is associated with phenolic compounds [54]. The 1,1-Diphenyl-2-picryl hydrazil (DPPH) radical scavenging activities of the NADES (70% LA:ChCl 3:1) extract of *F. vesiculosus* under the optimized conditions (Table 4) and 96% EtOH seaweed extracts increased in a concentration-dependent manner (Figure 3). Phloroglucinol as electron donor and as the structural unit of seaweed phlorotannins [55] was used as a standard antioxidant in this test. Phloroglucinol was effective in scavenging DPPH free radicals with an IC50 of 5.6 ± 0.5 μg/mL. The optimized NADES extract of *F. vesiculosus* was found to have free radical scavenging activity similar to that of phloroglucinol, especially at concentrations above 0.05 mg/mL. The DPPH radical scavenging activity of the EtOH extract of seaweed was equal to that of the NADES extract (Figure 4).

**Figure 5:**
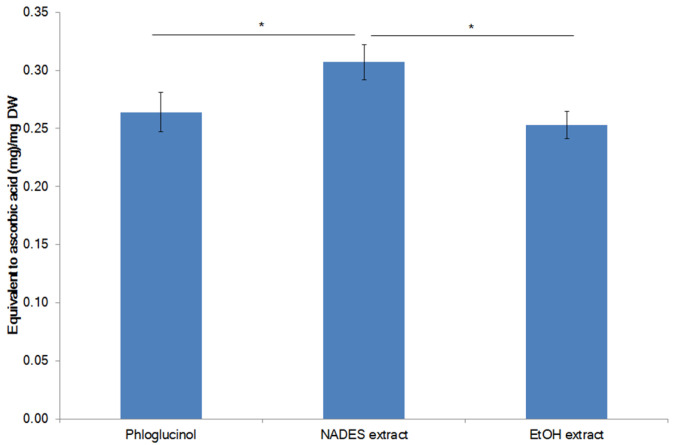
Total antioxidant capacity (TAC) of the *F. vesiculosus* extracts. * A significant difference (*p* < 0.05) was found between the phloroglucinol (reference compound) and the NADES extract, and between NADES extract and EtOH extract.

The total antioxidant capacity (TAC) of the *F. vesiculosus* extracts was analyzed in the reduction reaction of Mo(VI) to Mo(V) by the extracts studied, and was quantitatively expressed in equivalents of ascorbic acid (AscAE) [56]. The TAC for the NADES extract of *F. vesiculosus* obtained under the optimized conditions was 0.31 ± 0.02 mg AscAE/mg and was higher than that for the reference compound phloroglucinol (0.26 ± 0.02 mg AscAE/mg) and the EtOH extract (0.25 ± 0.01 mg AscAE/mg) (Figure 5).

According to our results, NADES (70% LA:ChCl 3:1) may be a valuable alternative for the efficient extraction of phlorotannins from the Arctic *F. vesiculosus* with strong antioxidant activity.

The IC50 for the purified ethyl acetate fraction of *Padina boergesenii* against the scavenging of DPPH radicals was 2.6 ± 1.2 mg/mL [14]. The phlorotannin-rich EtOH extract of *F. vesiculosus* obtained through pressurized liquid extraction inhibited DPPH radicals with an IC50 of 92.6 µg/mL [51]. The IC50 values for phlorotannins of the EtOH extract of *S. swartzii* in TAC and DPPH assays were 3.6 ± 0.5 mg ascorbic acid equivalents/g and 9.4 ± 0.5 mg/L, respectively [53]. Interestingly, in the current study, the antioxidant activity of the NADES extract obtained under the optimized conditions was equal to or higher than that of the EtOH *F. vesiculosus* extract (Figure 3 and Figure 4). To the best of our knowledge, this is the first study reporting the antioxidant activity of NADES *F. vesiculosus* extract. Our results are in line with other previous publications. The NADES extracts of *Polygonum maritimum* were found to be rich in phenolic compounds, and, therefore, were potent antioxidants in vitro. In particular, the NADES (choline chloride (ChCl) and sucrose (Suc)) extract was more efficient in the scavenging of DPPH radicals and oxygen radical absorbance capacity test, as compared to the acetone extracts [57]. The NADES extracts of onion peel prepared with ChCl, Suc, urea and sorbitol had a significant (2–5-fold) higher iron reduction capacity than that of the aqueous methanol extract. In the same study, the DPPH radical scavenging activity of the NADES extract (ChCl:sorbitol) was similar to that of the MeOH extract [58]. In general, our experimental and literature data confirm that NADES are a sustainable alternative to organic solvents in the extraction of polyphenols from various plant and seaweed species.

### 2.5. HPLC-HRMS Profiling of Phlorotannins in Extracts of F. vesiculosus

**Figure 6:**
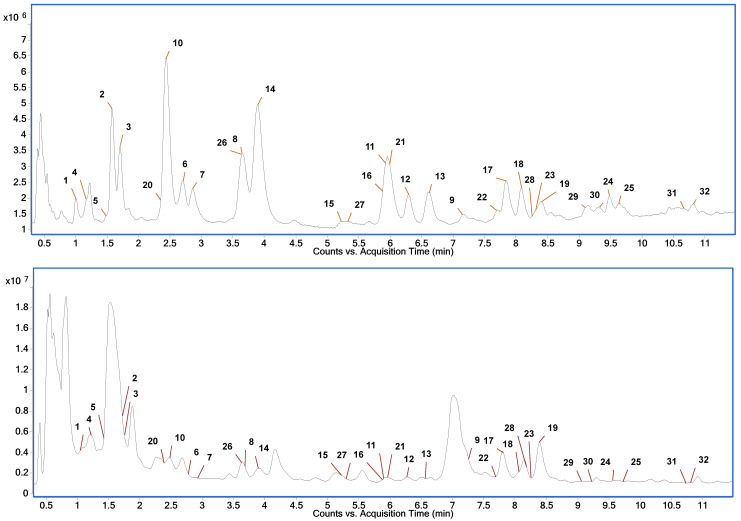
Typical total ion chromatogram of EtOH (**top**) and NADES (**bottom**) extracts of *F. vesiculosus*.

The EtOH and NADES extracts were analyzed through the HPLC-HRMS method to identify the phlorotannin composition of the extracts. Initially, the mass spectrometric data collected in a non-targeted manner were searched for the compounds for which the measured accurate values of the ratio of monoisotopic mass-to-charge (*m*/*z*) corresponded to the theoretical values of *m*/*z* for the deprotonated [M − H]^−^ molecules for the phlorotannins known from the literature [19,59,60,61,62]. Typical chromatograms of the EtOH and NADES extracts are presented in Figure 6. The selection took into account phlorotannins with a number of phloroglucinol monomeric units (PGU) ranging from 2 to 13. The extract ion chromatograms (EIC) recorded during the separation process contained the well-resolved peaks of the compounds that presumably correspond to phlorotannins with a number of PGU from 3 to 9. The elemental composition of the detected compounds was confirmed by measuring the exact mass with an error of no more than 5 ppm and by the nature of the isotopic distribution. Furthermore, preliminary identification of phlorotannins was carried out on the basis of the collision-induced dissociation (CID) spectra obtained as a result of targeted MS/MS experiments carried out for the detected compounds. The spectra recorded were studied and compared with the possible structures of phlorotannins based on the data described in the literature [21,59,60,61,62,63], the pattern of their fragmentation and the resulting product ions. The phlorotannins detected and identified in the extracts are presented in Table 5.

Both the ethanol and NADES extracts consisted of the same varieties of phlorotannins identified, mainly represented by oligomers containing from 5 to 9 PGUs. Based on the results of the search for polyphenols with three PGU units, only one compound detected (**1**, Table 5) was described in the literature [61], and was previously classified as fucophlorethol.

The CID spectra of two detected phlorotannins (**2**, **3**) consisting of four PGU units have similar fragmentation patterns (Table 5) and differ from each other mainly in the relative abundance of fragment ions. The spectra contain the peaks corresponding to the losses of a water molecule and one and two PGU units, including those in combination with a water molecule. In addition, the spectra contained the peaks of ions characterizing the fragments of fucol (*m*/*z* 229) and phloroglucinol (*m*/*z* 125). These patterns of fragmentation make it possible to tentatively identify two detected tetramers (**2**, **3**) as isomers of fucodiphlorethol [60,61].

The spectra of six detected pentamers (**4**–**9**) contained some fairly intensive peaks corresponding to the loss of one or two water molecules. Among these pentamers, compounds **4** and **5** clearly show the loss of only one unit of PGU upon fragmentation and are possibly trifucophlorethol isomers [60]. For the remaining pentamers (**6**–**9**), one to three PGU or their derivatives are already lost, including in combination with water. Thus, pentamers (**6**–**9**) were tentatively assigned to fucotriphlorethol isomers. This identification is in line with the literature data [60,61].

The spectral data of hexamer **10** were described earlier in the literature [64], and it can be tentatively identified as hexafucol based on the observed pattern of its fragmentation [52,59]. In the CID spectrum of phlorotannin **10**, there are ion peaks corresponding to the loss of one and two water molecules, which are characteristic of all the detected hexamers (**10**–**13**). Along with this, spectrum **10** shows no losses of phloroglucinol caused by breaking the ether bond, and the observed losses of 124 and 166 Da are apparently associated with breaks of aryl bonds and bonds in benzene rings [52,59]. The spectra of the other three hexamers (**11**–**13**) show a similar fragmentation pattern, resembling the spectra of the above-described fucophlorethols (**6**–**9**) with a difference of one PGU, and, taking into account the literature data [60], may be isomers of fucotetraphlorethol.

Compounds (**14**–**16**) can probably be assigned to heptafucol isomers, since their CID spectra correlate with the spectrum of hexafucol **10**, taking into account the difference by one PGU. The rest of the heptamers found (**17**–**19**) are most likely fucophlorethol isomers given the loss of one or two PGU. Further identification of these compounds is complicated by the structural diversity of phlorotannins and requires additional studies after isolating individual components. The same is valid for phlorotannins with a degree of polymerization of 8 and 9 PGU (compounds **20**–**32**), which were represented in the extracts by a fairly wide range of compounds, most of which can probably be attributed to fucophlorethol.

The identification of phlorotannins is too complicated due to the similar polarity and high variety of the polymer isomer structures [21,60,61,62]. Nevertheless, without future multistep purification, we were able to identify 32 phlorotannins (one trimer, two tetramers, six pentamers, four hexamers, six heptamers, six octamers and seven nonamers) in the Arctic *F. vesiculosus*. It was noticed that all the above-mentioned phlorotannins were identified in both the EtOH and NADES extracts.

In the current study, phlorotannins were extracted from the Arctic brown seaweed *F. vesiculosus*. Our results underlined the potential of NADES for the extraction of phlorotannins and evidence on perspectives of the green extraction of these compounds. The chemical profiling of NADES extracts provides a scientific basis for future pharmacological studies.

## 3. Materials and Methods

### 3.1. Materials

Fresh brown seaweed *Fucus vesiculosus* L. were collected from coastal regions of the Zelenetskaya Bay of the Barents Sea (Russia) in April 2022. The seaweed samples were identified by Dr. E. Obluchinskaya, and voucher specimens (No. 4.2022) were deposited in the Collection of the Zoobentos Laboratory, Murmansk Marine Biology Institute. The seaweed samples then were cleaned from epiphytes by gently rubbing their surface after washing twice in filtered seawater. The materials were frozen and kept at −25 °C.

L-Lactic acid (88.0–92.0% CAS 79-33-4) was from Panreac Química SLU (Barcelona, Spain). Choline chloride (>98.0% CAS 67-48-1) was purchased from Acros Organics (Fair Lawn, NJ, USA). These chemicals were used as received. Folin–Ciocalteu reagent, phloroglucinol and 2,2-diphenyl-1-picrylhydrazyl (DPPH) were from Sigma-Aldrich (St. Louis, MO, USA). Water was purified using a Milli-Q system (Millipore, Bedford, MA, USA). Other analytical-grade chemicals and solvents for extraction and assays were purchased from local chemical suppliers.

### 3.2. NADES Preparation

The NADES were synthesized using a heating technique [49]. The molar ratios of lactic acid and the hydrogen bond donor choline chloride were 3:1, according to our previous study [45]. A bottle containing the pre-weighed ingredients was heated in a water bath at 50 °C for 60 min with agitation at 700 rpm until a clear liquid formed. The liquid was placed under vacuum in an RI-1 rotary evaporator (Ecroskhim Ltd., Moscow, Russia) at 40 °C, cooled and stored in sealed glass vials in a dark place at 20 ± 2 °C. The NADES prepared were transparent liquids that were stable during storage. The water content in NADES was determined using the Karl Fischer method and was 4.1%. NADES were diluted with water based on the certain percentage of water [65].

### 3.3. Extraction of Phlorotannins with NADES and EtOH

*F. vesiculosus* frozen samples were broken up into small pieces of 1–3 mm^2^, thawed at room temperature and then mixed with the solvent in a proportion determined using the experimental design matrix. The Branson MT-3510 ultrasonic bath (Branson Ultrasonics Corporation, Danbury, CT, USA) was used for the ultrasound-assisted extraction (42 kHz and 130 W) of seaweed samples with NADES. After the samples were centrifuged at 3000× *g* for 15 min at 20 °C, a liquid layer was employed for further analysis. The EtOH extract was prepared through percolation at a seaweed:solvent ratio of 1:10 at 20 °C in the dark.

### 3.4. Experimental Design and Statistical Analysis

A three-level, three-variable BBD was employed in this study. The design consisted of 15 experimental runs. The parameters as well as the coded and original values of the independent variables used in this experiment are presented in Table 1. The total phlorotannin content (TPhC, mg TPHC/g DW) from the BBD was analyzed using the response surface regression (Table 2) procedure of the statistical analysis system (STATGRAPHICS Centurion XV) and fitted to a second-order polynomial model, as shown in Equation (2).

(2) $$Y=\beta_{0}+\sum_{i=1}^{k}\beta_{i}X_{i}+\sum_{i=1}^{k}\beta_{ii}X_{i}^{2}+\sum \sum_{j}^{k}\beta_{ij}X_{i}X_{j}$$

where Y is the predicted response (TPhC), β_0_ is the constant coefficient, β_i_ is the linear coefficient, β_ii_ is the quadratic coefficient, β_ij_ is the cross-product coefficients and X_i_ and X_j_ are independent variables. Two-dimensional contour plots were developed while keeping one variable constant in the second-order polynomial model. The validity of the model was determined by comparing the experimental and predicted values.

Statistical analysis was performed using STATGRAPHICS Centurion XV (StatPoint Technologies Inc., Warrenton, VA, USA) statistical software. Data are presented as mean values ± standard deviations. The comparison of quantitative variables was performed using analysis of variance (ANOVA), and the differences were calculated using Duncan’s test (*p* < 0.05).

### 3.5. The Phlorotannin Content

The spectrophotometry method [66,67] was used for the analysis of total phlorotannin content (TPhC) in *F. vesiculosus* extracts. Phloroglucinol was used as a reference compound. Briefly, after mixing 100 L of the extract or phloroglucinol with 2 mL of 2% Na_2_CO_3_, 100 µL of the Folin–Ciocalteu reagent was added. The mixture was incubated for 30 min at room temperature and in the dark. The absorbance of the reaction was measured at 720 nm using a spectrophotometer Shimadzu UV 1800 (Shimadzu, Kyoto, Japan). The TPhC was expressed as mg phloroglucinol equivalent per gram of dry weight (mg PhE/g DW) of seaweed and expressed as the mean ± SD of three independent experiments performed in duplicate.

### 3.6. The Antioxidant Activity

The DPPH radical scavenging activity was determined according to a well-known procedure [54]. In brief, 50 µL test solution was mixed with 50 µL methanol and DPPH solution (100 µL), vortexed and kept in the dark at room temperature for 30 min. The decrease in absorbance of the mixture was measured at 517 nm against a reagent blank. Phloroglucinol was used as the reference standard. All measurements were performed in duplicate.

The total antioxidant capacity (TAC) of the fucoidan-containing samples was evaluated as described [56]. Briefly, in duplicate, an aliquot of 0.1 mL of NADES extract obtained using the optimized parameters and EtOH extract (pre-diluted 1:100 in water) was combined with 1 mL of reagent (0.6 M sulfuric acid, 28 mM sodium phosphate and 4 mM ammonium molybdate). The tubes were incubated at 95 °C for 90 min. After cooling the samples, their absorbance was recorded on a Shimadzu UV 1800 spectrophotometer (Shimadzu, Kyoto, Japan) at 695 nm against a blank. The blank solution contained 1 mL reagent solution and the appropriate volume of water. Phloroglucinol was used as a reference standard. Ascorbic acid was used as a standard for the calibration curve. TAC was expressed in mg equivalents of ascorbic acid per mg sample (AscAE/mg).

### 3.7. HPLC-HRMS Analysis

#### 3.7.1. Sample Preparation

The EtOH extract of phlorotannins was concentrated in a vacuum rotary evaporator at a temperature of 30 °C until the alcohol was removed. The resulting concentrate was diluted with a 0.1% aqueous solution of formic acid in a ratio of 1:3. The aqueous mixture was applied to a “Mega Bond Elut C18” cartridge (1 g sorbent per cartridge) (part number 12256001, Agilent, Santa Clara, CA, USA), which was pre-washed with acetonitrile and then with 0.1% aqueous formic acid. After applying the sample, the cartridge was washed with 3 volumes of 0.1% aqueous formic acid. The phlorotannins were eluted with acetonitrile containing 0.1% formic acid, and the eluate was evaporated in a stream of nitrogen at 30 °C until dry, and then reconstituted in a 0.1% aqueous solution of formic acid. The resulting solution was filtered through a porous filter with a pore size of 0.22 μm (Agilent) before analysis.

A sample of the NADES extract was diluted with acetonitrile in a ratio of 1:5, and the resulting mixture was shaken and centrifuged for 5 min at 10,000 rpm. The supernatant was separated and diluted with 0.1% aqueous formic acid to the required concentration for analysis.

#### 3.7.2. HPLC-HRMS and MS/MS Analysis

HPLC-HRMS and MS/MS analysis of the compounds in both extracts was performed on an Agilent 1290 Infinity II HPLC system connected with a quadrupole time-of-flight (Q-TOF) accurate mass detector (Agilent 6545 Q-TOF LC-MS, Agilent Technologies, Santa Clara, CA, USA). Chromatographic separation was performed using a “Zorbax Eclipse Plus C18” (2.1 mm × 50 mm, 1.8 μm, Agilent Technologies, p/n 959757-902) column with an additional 5 mm guard column. The column thermostat temperature was 30 °C. The mobile phase is composed of solvent A, containing 0.1% (*v*/*v*) formic acid in water, and solvent B, consisting of 0.1% formic acid (*v*/*v*) in acetonitrile. Gradient elution was carried out according to the following program: the initial concentration of solvent B was kept at 5% for 4 min, then the concentration of this solvent changed linearly up to 40% by 24 min, then up to 100% by 34 min with an exposure for 2 min. Flow rate was kept at 0.35 mL/min.

The injection volume was 1 μL. The Q-TOF instrument was operated with an electrospray source in negative ion mode using the following conditions: drying gas temperature, 350 °C (nitrogen, 10 L/min); nebulizer pressure, 40 psi; capillary voltage, 4000 V and fragment or voltage, 100 V. In the MS/MS mode, the quadrupole was adjusted to isolate precursor ions with a bandwidth of Δ *m*/*z* = 1.3. The CID spectra of the precursor ions were recorded with collision energy (CE) in the range of 20–50 eV. The collision cell was filled with nitrogen (99.999%). Ions were scanned in an *m*/*z* range of 100–1700. The TOF detector was operated in EDR (2 GHz) mode, and the acquisition was 1.5 spectra/s. The mass spectrometer was adjusted, and the mass measurement accuracy was corrected automatically in accordance with the instruction manual of the device and using recommended standard solutions (Agilent, part. numbers G1969-85000 and G1969-85001).

## 4. Conclusions

In the current study, a fast and green NADES-UAE procedure was developed for the extraction of phlorotannins from Arctic brown seaweeds *F. vesiculosus*. Process optimization using the BBD and RSM approaches resulted in a NADES extract with high phlorotannin content (137.3 mg TPhC/g DW). The antioxidant activity of the optimized NADES extract was equal to that of the EtOH extract.

As far as we know, this is the first study in which the chemical profiling of phlorotannins in a NADES extract was performed. Using HPLC-HRMS and MS/MS analyses, we were able to identify 32 phlorotannins (one trimer, two tetramers, six pentamers, four hexamers, six heptamers, six octamers and seven nonamers) in the NADES extract of *F. vesiculosus*. It was noted that all the above-mentioned phlorotannins were identified in both the EtOH and NADES extracts.

Our results suggests that NADES lactic acid:choline chloride (3:1) could be considered as an alternative solvent for the effective extraction of phlorotannins from *F. vesiculosus* with high antioxidant potential.

## Figures and Tables

**Table 1:** Factors and their coded levels used in the Box–Behnken design for UAE extraction of *F. vesiculosus* with NADES.

Factors	Levels		
	−1	0	+1
Time (min), X_1_	10	20	30
Water concentration in NADES (%), X_2_	30	40	50
Sample to solvent ratio (g/g), X_3_	7.5	10.0	12.5

**Table 2:** Box–Behnken experimental design matrix and the experimental and predicted values observed for TPhC.

Run	Independent Variables (Factors)			Total Phlorotannin Content (mg PhE/g DW)	
	X_1_	X_2_	X_3_	Experimental	Predicted
1	1	−1	0	54.2 ± 0.6	50.8
2	0	0	0	60.3 ± 2.9	60.2
3	−1	−1	0	22.0 ± 0.3	20.9
4	0	−1	−1	44.7 ± 2.8	47.1
5	1	0	1	80.4 ± 2.0	81.6
6	0	1	−1	73.8 ± 4.6	71.7
7	0	0	0	60.4 ± 0.1	60.2
8	−1	1	0	52.3 ± 2.8	55.8
9	1	0	−1	51.1 ± 1.1	52.1
10	−1	0	1	43.7 ± 3.2	42.6
11	1	1	0	78.1 ± 1.7	79.2
12	0	0	0	60.0 ± 3.6	60.2
13	−1	0	−1	39.2 ± 1.4	37.9
14	0	1	1	98.2 ± 2.9	95.8
15	0	−1	1	55.0 ± 2.0	57.1

**Table 3:** ANOVA analysis of the response surface quadratic model for optimizing the total phlorotannin content.

Source	Sum of Squares	df	Mean Square	*F*-Value	*p*-Value
Model	4864.01	9	540.446	51.86	0.0002
X_1_	1416.98	1	1416.98	135.98	0.0001
X_2_	1999.02	1	1999.02	191.84	0.0000
X_3_	585.675	1	585.675	56.20	0.0007
X_1_X_2_	10.3684	1	10.3684	1.00	0.3643
X_1_X_3_	152.646	1	152.646	14.65	0.0123
X_2_X_3_	50.1264	1	50.1264	4.81	0.0798
$X_{1}^{2}$	486.01	1	486.01	46.64	0.0010
$X_{2}^{2}$	31.0434	1	31.0434	2.98	0.1449
$X_{3}^{2}$	85.6774	1	85.6774	8.22	0.0351

**Table 4:** The optimized parameters used in the extraction process and comparative concentration of TPhC of the predicted and experimental results.

Optimal Parameters			Predicted Results (mg PhE/g DW)	Experimental Results (mg PhE/g DW)
Extraction Time (min)	Water Concentration (%)	Sample to Solvent Ratio (m/m)		
22.8	30.0	1.0:12.0	140.3	137.3 ± 2.9

**Table 5:** Phlorotannins detected in EtOH and NADES extracts of *F. vesiculosus*.

DP	Molecular Formula	No.	t*_R_* (min)	MS/MS (CID) Spectrum *	Tentative Identification
Trimer	C_18_H_14_O_9_	1	1.02	355.0455 (-H_2_O), 329.0659, 305.0663, 287.0560, 261.0770, 247.0229 (-1PGU), 243.0662, 231.0294 (-1PGU, -H_2_O, +2), 216.0057, 205.0500, 189.0553, 165.0191, 149.0240, 141.0189, 124.0163	Fucophlorethol
Tetramer	C_24_H_18_O_12_	2	1.57	479.0613 (-H_2_O), 453.0817, 435.0708, 371.0401 (-1PGU), 353.0297 (-1PGU, -H_2_O), 339.0504, 335.0191 (-1PGU, -2H_2_O), 309.0397, 247.0240 (-2PGU, +2), 229.0145 (-2PGU, -H_2_O, +2), 219.0299, 139.0032, 125.0240	Fucodiphlorethol
		3	1.71	479.0606 (-H_2_O), 453.0813, 371.0399 (-1PGU), 353.0298 (-1PGU, -H_2_O), 339.0504, 335.0195 (-1PGU, -2H_2_O), 313.0711, 309.0399, 265.0357, 247.0238 (-2PGU, +2), 229.0131 (-2PGU, -H_2_O, +2), 139.0030, 125.0232	Fucodiphlorethol
Pentamer	C_30_H_22_O_15_	4	1.20	603.0763 (-H_2_O), 585.0653 (-2H_2_O), 577.0957, 559.0865, 495.0561 (-1PGU), 477.0453 (-1PGU, -H_2_O), 455.0612, 433.0554, 331.0452, 311.0196, 289.0346, 267.0299, 247.0241, 229.0136, 207.0291, 165.0190, 139.0030, 125.024	Trifucophlorethol
		5	1.49	603.0769 (-H_2_O), 585.0662 (-2H_2_O), 577.0974, 559.0868, 535.0865, 495.0556 (-1PGU), 477.0455 (-1PGU, -H_2_O), 455.0618, 433.0545, 413.0497, 329.0294, 311.0185, 289.0358, 267.0293, 245.0092, 229.0130, 207.0301, 165.0190, 139.0032, 125.0234	Trifucophlorethol
		6	2.74	603.0769 (-H_2_O), 585.0666 (-2H_2_O), 559.0870, 495.0558 (-1PGU), 477.0456 (-1PGU,-H_2_O), 461.0508, 371.0405 (-2PGU, +2), 353.0297 (-2PGU, -16/-2PGU,-H_2_O, +2), 335.0194 (-2PGU, -2H_2_O, +2), 309.0397, 249.0400, 229.0137 (-3PGU, -H_2_O, +4), 139.0033, 125.0240	Fucotriphlorethol
		7	2.89	603.0777 (-H_2_O), 585.0641 (-2H_2_O), 559.0869, 495.0562 (-1PGU), 477.0454 (-1PGU, -H_2_O), 371.0397 (-2PGU, +2), 353.0297 (-2PGU, -16/-2PGU,-H_2_O, +2), 335.0190 (-2PGU, -2H_2_O, +2), 309.0399, 249.0397, 229.0138 (-3PGU, -H_2_O, +4), 139.0030, 125.0240	Fucotriphlorethol
		8	3.68	603.0770 (-H_2_O), 585.0660 (-2H_2_O), 559.0867, 495.0565 (-1PGU), 477.0457 (-1PGU,-H_2_O), 371.0409 (-2PGU, +2), 353.0298 (-2PGU, -16/-2PGU, -H_2_O, +2), 339.0507, 337.0350, 335.0191 (-2PGU, -2H_2_O, +2), 245.0088 (-3PGU, +2), 229.0139 (-3PGU, -H_2_O, +4), 139.0032, 125.0241	Fucotriphlorethol
		9	7.16	603.0753 (-H_2_O), 495.0552 (-1PGU), 477.0444 (-1PGU, -H_2_O), 459.0354 (-1PGU, -2H_2_O), 389.0505, 371.0403 (-2PGU, +2), 353.0300 (-2PGU, -16/-2PGU, -H_2_O, +2), 337.0337, 263.0190, 247.0240, 245.0087 (-3PGU, +2), 229.0140 (-3PGU, -H_2_O, +4), 139.0029, 125.0240	Fucotriphlorethol
Hexamer	C_36_H_26_O_18_	10	2.45	727.0931 (-H_2_O), 709.0823 (-2H_2_O), 683.1031, 585.0669, 579.0768, 455.0615, 437.0511, 411.0714, 289.0348, 165.0189, 139.0032	Hexafucol
		11	5.96	727.0933 (-H_2_O), 709.0825 (-2H_2_O), 619.0745 (-1PGU), 601.0626 (-1PGU, -H_2_O), 583.0513, 477.0456 (-2PGU, -16/-2PGU, -H_2_O, +2), 461.0522, 373.0557, 353.0295, 335.0189, 245.0086, 229.0134, 139.0034	Fucotetraphlorethol
		12	6.31	727.0925 (-H_2_O), 709.0803 (-2H_2_O), 619.0707 (-1PGU), 477.0451 (-2PGU, -16/-2PGU, -H_2_O, +2), 371.0401, 353.0298, 337.0343, 247.0238, 229.0139, 139.0033	Fucotetraphlorethol
		13	6.61	727.0924 (-H_2_O), 709.0797 (-2H_2_O), 601.0605 (-1PGU, -H_2_O), 583.0512, 477.0461 (-2PGU, -16/-2PGU, -H_2_O, +2), 337.0345, 245.0091, 229.0138, 139.0030	Fucotetraphlorethol
Heptamer	C_42_H_30_O_21_	14	3.92	851.1090 (-H_2_O), 833.0983 (-2H_2_O), 708.0750, 703.0936, 579.0773, 455.0614, 437.0516, 413.0511, 289.0351, 269.0088, 165.0191, 139.0033, 125.0242	Heptafucol
		15	5.26	851.1085 (-H_2_O), 833.0976 (-2H_2_O), 703.0940, 579.0783, 455.0601, 413.0508, 289.0356, 139.0030	Heptafucol
		16	5.90	851.1086 (-H_2_O), 833.1003 (-2H_2_O), 579.0775, 289.0349, 139.0030	Heptafucol
		17	7.86	851.1089 (-H_2_O), 727.0934, 725.0766 (-1PGU, -H_2_O), 707.0670 (-1PGU, -2H_2_O), 619.0771 (-2PGU, +2), 601.0614 (-2PGU, -16/-2PGU, -H_2_O, +2), 583.0501, 497.0720, 479.0626, 461.0512, 371.0404, 353.0300, 335.0192, 247.0235, 229.0138, 139.0033	Fucophlorethol
		18	8.09	851.1090 (-H_2_O), 725.0770 (-1PGU, -H_2_O), 707.0676 (-1PGU, -2H_2_O), 601.0609 (-2PGU, -16/-2PGU, -H_2_O, +2), 461.0515, 371.0399, 353.0299, 335.0198, 229.0137	Fucophlorethol
		19	8.41	851.1081 (-H_2_O), 725.0769 (-1PGU, -H_2_O), 707.0659 (-1PGU, -2H_2_O), 477.0452, 461.0515, 371.0403, 353.0301, 337.0349, 229.0139	Fucophlorethol
Octamer	C_48_H_34_O_24_	20	2.36	975.1219 (-H_2_O), 957.1110 (-2H_2_O), 939.1012, 745.1023, 727.0914, 708.0727, 455.0608, 289.0341, 247.0238, 229.0140	Octafucol
		21	5.99	975.1245 (-H_2_O), 957.1143 (-2H_2_O), 867.1024 (-1PGU), 849.0925 (-1PGU, -H_2_O), 832.0905 707.0670 692.0798 579.0777 454.0540 413.0514 287.0198 269.0089 165.0192 125.0243	Fucophlorethol
		22	7.70	975.1239 (-H_2_O), 957.1132 (-2H_2_O), 832.0891, 703.0926, 579.0769, 413.0509, 289.0350, 287.0196, 269.0092, 139.0030, 125.0243	Octafucol
		23	8.31	975.1227 (-H_2_O), 850.1005 727.0926 603.0744, 477.0458, 461.0503, 372.0482, 353.0287, 338.0435, 247.0248, 229.0141, 139.0039	Octafucol
		24	9.48	975.1236 (-H_2_O), 849.0921 (-1PGU, -H_2_O), 831.0824 (-1PGU, -2H_2_O), 601.0619, 583.0514, 495.0571, 477.0454, 461.0514, 353.0305, 335.0195, 229.0141, 139.0035	Fucophlorethol
		25	9.64	975.1237 (-H_2_O), 849.0922 (-1PGU, -H_2_O), 831.0825 (-1PGU, -2H_2_O), 725.0769, 495.0568, 477.0463, 461.0508, 371.0408, 353.0305, 335.0195, 245.0088, 229.0141, 139.0034	Fucophlorethol
Nonamer	C_54_H_38_O_27_	26	3.64	1099.1391 (-H_2_O), 1081.1257 (-2H_2_O), 1063.1158, 869.1167, 851.1078, 832.0883, 579.0775, 519.0556, 413.0492, 287.0180, 247.0240, 229.0130	Nonafucol
		27	5.33	1099.1422 (-H_2_O), 1081.1278 (-2H_2_O), 1063.1163, 956.1099, 938.0925, 809.0996, 745.1041, 725.0770, 708.0751, 643.0702, 555.0580, 455.0609, 353.0298, 289.0365, 229.0149	Nonafucol
		28	8.25	1099.1397 (-H_2_O), 1081.1279 (-2H_2_O), 955.0955 (-1PGU, -2H_2_O), 851.1070, 727.0926, 603.0767, 477.0472, 371.0412, 353.0302, 247.0247, 229.0142	Fucophlorethol
		29	9.11	1099.1403 (-H_2_O), 1081.1309 (-2H_2_O), 956.1074, 827.1070, 703.0928, 455.0607, 413.0512, 287.0194, 269.0090, 247.0247, 229.0144, 165.0181, 139.0031	Nonafucol
		30	9.34	1099.1389 (-H_2_O), 1081.1278 (-2H_2_O), 973.1061 (-1PGU, -H_2_O), 956.1036, 827.1058, 725.0738, 708.0724, 455.0610, 373.0562, 289.0340, 269.0090, 247.0238, 229.0133, 165.0186, 139.0032	Fucophlorethol
		31	10.67	1099.1405 (-H_2_O), 973.1087 (-1PGU, -H_2_O), 849.0923 (-2PGU, -16/-2PGU, -H_2_O, +2), 835.1120, 727.0931, 709.0806, 601.0609, 461.0519, 353.0299, 229.0142, 139.0028	Fucophlorethol
		32	10.82	1099.1398 (-H_2_O), 973.1063 (-1PGU, -H_2_O), 955.0959 (-1PGU, -2H_2_O), 849.0919 (-2PGU, -16/-2PGU, -H_2_O, +2), 745.1022, 725.0747, 601.0600, 495.0559, 477.0463, 353.0303, 229.0142, 139.0035	Fucophlorethol

DP—degree of polymerization; t*_R_*—retention time. * Main characteristic product ions.

## Data Availability

The data are available on request from the corresponding author.
